# Milk Production, Composition, and Fatty Acid Profile in Milk from Dairy Cows Fed Increasing Levels of Dietary Soybean Oil: A Dose-Response Study

**DOI:** 10.3390/vetsci13050456

**Published:** 2026-05-07

**Authors:** Yanitl Citlali Acho-Martínez, Pedro Abel Hernández-García, Enrique Espinosa-Ayala, Ofelia Márquez-Molina, Germán David Mendoza-Martínez, Gabriela Vázquez-Silva, Pablo Benjamín Razo-Ortiz, Cesar Diaz-Galván, José Felipe Orzuna-Orzuna

**Affiliations:** 1 Centro Universitario Amecameca, Universidad Autónoma del Estado de México, Amecameca CP 56900, Mexico; yanincitlaliancho@gmail.com (Y.C.A.-M.); pedro_abel@yahoo.com (P.A.H.-G.); enresayaa1@hotmail.com (E.E.-A.); ofeliamarquez11@hotmail.com (O.M.-M.); mvzrazo@gmail.com (P.B.R.-O.); 2 Departamento de Producción Agrícola y Animal, Universidad Autónoma Metropolitana—Xochimilco, Mexico City CP 04960, Mexico; gmendoza@correo.xoc.uam.mx (G.D.M.-M.); cesarwardi14@gmail.com (C.D.-G.); 3 Departamento del Hombre y su Ambiente, Universidad Autónoma Metropolitana—Xochimilco, Mexico City CP 04960, Mexico; gavaz@correo.xoc.uam.mx; 4 Unidad Regional Universitaria de Zonas Áridas, Universidad Autónoma Chapingo, Bermejillo CP 35230, Mexico

**Keywords:** vegetable oil, milk yield, milk quality, Holstein cows

## Abstract

The objective of this study was to evaluate the effects of dietary inclusion of increasing levels of soybean oil on milk yield, milk composition, and milk fatty acid profile in dairy cows. The experiment was designed as a 4 × 4 double Latin square with 21-day periods and used eight Holstein cows (body weight of 550 ± 19.5 kg and 200 ± 5 days in milk). The treatments evaluated were a basal diet without soybean oil and a basal diet added with 10, 20, or 30 g/kg DM of soybean oil. None of the treatments evaluated affected (*p* > 0.05) milk yield, dry matter intake, dry matter digestibility, or the concentration of fat, non-fat solids, protein, and lactose in milk. Furthermore, increasing levels of soybean oil included in the diets did not affect (*p* > 0.05) the milk concentration of butyric, caproic, caprylic, capric, lauric, pentadecanoic, palmitoleic, heptadecanoic, and linoleic fatty acids. However, dietary inclusion of soybean oil decreased (linear effect; *p* ≤ 0.05) the milk concentration of myristic, palmitic and oleic fatty acids. In contrast, higher concentrations (linear effect; *p* ≤ 0.05) of stearic fatty acids were observed in the milk of dairy cows fed soybean oil in their diet. In conclusion, dietary inclusion of increasing levels of soybean oil (30 g/kg DM) modifies the fatty acid profile of milk without affecting milk yield or the protein, fat, or lactose content.

## Simple Summary

The chemical composition and fatty acid profile of dairy cow milk can be modified through the inclusion of vegetable oils in the diet. The inclusion of increasing levels of soybean oil in dairy cow diets did not affect milk yield, dry matter intake, dry matter digestibility, or milk protein, fat, and lactose content. However, the inclusion of moderate levels (30 g/kg DM) of soybean oil in dairy cow diets modifies the fatty acid profile in milk. In conclusion, the inclusion of soybean oil in dairy cow diets modifies the fatty acid profile of milk without affecting milk production or the protein, fat, or lactose content.

## 1. Introduction

According to Godina-Rodríguez et al. [1], a growing global demand (+35%) for ruminant milk is expected to meet the needs of the increasing human population. Ruminants also play a key role in converting non-edible biomass into nutrient-dense foods contributing to food system sustainability [2]. Although ruminant milk contains nutrients beneficial to consumers, it also has a significant concentration of saturated fatty acids (SFAs) [3]. This intrinsic characteristic of ruminant milk is undesirable because, according to Orzuna-Orzuna et al. [4], high intake of SFAs is associated with a higher incidence of cardiovascular disease (CVD). Consequently, milk consumers have shown increased interest in purchasing products with low SFA content and, preferably, high polyunsaturated fatty acid (PUFA) content [5]. In contrast to SFAs, PUFAs have been associated with a lower incidence of stroke and heart disease [6]. Therefore, it is necessary to evaluate nutritional strategies that can improve the fatty acid profile of ruminant milk.

In recent years, several novel strategies have been evaluated to improve the fatty acid profile in ruminant milk, such as dietary supplementation with microalgae [4] and products derived from *Cannabis sativa* seeds [1]. However, these strategies require extensive evaluation of their potential risks to human health before large-scale implementation. Recent approaches have focused on improving nutrient efficiency and sustainability through balanced protein-energy strategies and alternative feed resources [7]. Other nutritional strategies, such as the use of plant-derived bioactive compounds, have also been explored for their capacity to modulate rumen fermentation and improve efficiency [8,9]. On the other hand, the dietary inclusion of vegetable oils in dairy ruminant diets has been used for several years as a nutritional strategy to improve milk composition and fatty acid profile [10]. Soybean, canola, and flaxseed oils are among the most frequently evaluated vegetable oils in dairy cow feed [11,12,13]. However, some recent meta-analyses [14,15] reported that the effects of vegetable oils on the composition and fatty acid profile of dairy cow milk have been inconsistent, requiring further studies to clarify the direction (positive or negative) of the treatment effect.

Dietary inclusion of high (50 g/kg DM) and moderate (36 g/kg DM) doses of soybean oil has been reported to decrease milk fat content [16,17], without altering milk yield or protein and lactose content in dairy cows’ milk. In contrast, Zheng et al. [18] observed no significant changes in milk yield or in the fat, protein, and lactose content of milk from dairy cows fed low doses (20 g/kg DM) of soybean oil in their diet. However, the three studies cited above [16,17,18] reported that dietary inclusion of soybean oil decreases SFAs in milk while increasing PUFA concentration. These findings suggest that some effects of soybean oil in dairy cows are dose dependent. The hypothesis of the present study is that increasing levels of soybean oil in dairy cow diets will positively modify milk yield, milk composition, and the fatty acid profile. Therefore, the objective of this study was to evaluate the effects of dietary inclusion of increasing levels of soybean oil on milk yield, milk composition, and milk fatty acid profile of dairy cows.

## 2. Materials and Methods

### 2.1. Design, Animals, and Diet

The experimental phase of the current study was conducted at Autonomous University of the State of Mexico. The Research Ethics and Bioethics Committee of the Autonomous University of the State of Mexico evaluated and approved the experimental procedures applied to the dairy cows used in the current study, through Protocol #22-11-2023 (unique identification code 24188-C-8).

The experiment was designed as a 4 × 4 double Latin square with 21-day periods and used eight Holstein cows (body weight of 550 ± 19.5 kg and 200 ± 5 days in milk). Four 21-day periods were used throughout the experiment. In each experimental period, 17 days were used for adaptation to the diets, and the last four days (days 18 to 21) were used to measure milk yield and dry matter intake, and milk samples (to measure milk composition and fatty acids) and fecal samples (to measure digestibility) were taken. The treatments evaluated were a basal diet without soybean oil and a basal diet supplemented with 10, 20, or 30 g/kg DM of soybean oil. The intermediate soybean oil dose (20 g/kg DM) used in the current study was selected based on the results reported by Zheng et al. [18]. These authors [18] observed that the inclusion of soybean oil at a moderate dose (20 g/kg DM) increased PUFA concentration and decreased SFA concentration in milk without affecting milk composition or productive parameters of dairy cows. However, some recent meta-analyses [14,15] have reported that vegetable oils have dose-dependent effects on milk production and composition in dairy cows. Consequently, the current study included both a lower (10 g/kg DM) and a higher (30 g/kg DM) soybean oil dose. The cows were housed in individual pens with ad libitum access to fresh water. The dairy cows were fed twice daily (7:30 and 17:30 h) ad libitum, with 10% more feed offered each day than the amount consumed the previous day.

The diets were prepared using traditional ingredients from the region where the study was conducted [19]. These diets were formulated based on the requirements established by the NRC [20] for dairy cows. The nutritional composition of the diets (Table 1) of the dairy cows was evaluated following the procedures described by AOAC [21] and Van Soest et al. [22].

### 2.2. Milk Yield, Milk Composition, and Fatty Acid Profile in Milk

During the experimental phase, dairy cows were milked twice daily at 7:00 a.m. and 5:00 p.m. using a mechanical milking machine. Milk yield (kg/d) from each cow was measured during the last four days of each experimental period using 30 kg capacity milk meters (Waikato MK, New Zealand) with a precision of 100 mL, as previously reported by Granados-Rivera et al. [23]. Additionally, milk samples (250 mL/day) were collected from each cow at each milking and refrigerated at 5 °C until analysis [24]. Dry matter intake (DMI, kg/d) was estimated as daily feed offered minus feed refused. During the last four days of each experimental period (days 18 to 21), fecal samples were taken directly from the rectum of each cow at 06:30 and 16:30 h. These samples were used to estimate the diet’s dry matter digestibility (DMD), using acid-insoluble ash as an internal marker and following the procedures described in detail by Van Keulen and Young [25].

The fat, non-fat solids, protein, and lactose content of milk were measured in triplicate (using a composite sample each of the four sampling days) using a Milkoscan^®^-FT120 automated analyzer (Milkoscan, Foss Electric, Hillerød, Denmark) [26]. The fatty acid profile of milk was measured in triplicate using 50 µL of lipid extracts from the milk, following the procedures described in detail by Granados-Rivera et al. [23] and the methylation method described in detail by Candia-López et al. [24]. Briefly, the milk samples were treated with sodium methoxide (0.5 M in methanol). Subsequently, the samples were vortexed and heated to 80 °C for 10 min. Finally, the samples were cooled, and the lipids were extracted with hexane and potassium carbonate. After drying and filtration (using a 0.45 µm nylon membrane) of the samples, fatty acid methyl esters (FAMEs) were determined using a Hewlett-Packard 6890 gas chromatograph (Hewlett-Packard 6890, Camarillo, CA, USA). The gas chromatograph used for the FAME analysis contained a Sp-2560 column (100 m × 0.25 mm × 0.20 µm), and the oven temperature was set from 100 °C to 235 °C (5 °C/min), while helium gas was used as the carrier (32 cm/s). Finally, the fatty acids in the milk were identified by their retention times relative to those of a standard mixture (Nu-Check Prep, Nu-Check, Reston, VA, USA).

### 2.3. Statistical Analysis

All data were analyzed using a 4 × 4 double Latin square design, through the PROC MIXED procedure of the Statistical Analysis System (SAS, Version 9.3, Raleigh, NC, USA) software [27], as reported by other authors for studies with this type of experimental design [28,29]. Furthermore, prior to statistical analysis, the normality and homoscedasticity of the data were verified using the SAS statistical software [27]. The date of access to the statistical software for data analysis was 26 September 2025. The main reason for using this experimental design is to control variability between cows and periods using a small number of animals. The double Latin Square design allows each cow to receive all treatments at different times. This enables the evaluation of the four soybean oil levels with greater precision and efficiency using only eight cows. Additionally, since equally spaced increasing doses of soybean oil were used (dose-response experiment), polynomial orthogonal contrasts were applied to evaluate the linear and quadratic effects of the inclusion levels (0, 1.0, 2.0, and 3.0% DM of diet) of soybean oil in the diet, as suggested by Kaps and Lamberson [30] for dose-response experiments. The complete statistical model used in the data analysis was:

Y_ijklm_ = µ + T_i_ + QL_j_ + A_(j)k_ + P_(j)l_ + ε_ijklm_,

In this model, Y_ijklm_ = dependent variable; µ = overall mean; T_i_ = fixed effect of treatment; QL_j_ = random effect of Latin Square; A_(j)k_ = random effect of animal within Latin Square; P_(j)l_ = random effect of period within Latin Square, and ε_ijklm_ = random error. The interaction between treatment and period was not significant for all variables and was removed from the statistical model. Statistical differences between treatments were considered when *p* < 0.05. Tukey’s test was used to compare the means between treatments.

## 3. Results

### 3.1. Milk Yield and Milk Composition

Table 2 shows that dietary inclusion of increasing levels (10, 20, and 30 g/kg DM) of soybean oil did not affect (*p* > 0.05) milk yield (MY), dry matter intake (DMI), dry matter digestibility (DMD), and concentration of fat, non-fat solids, protein, and lactose in the milk of dairy cows.

### 3.2. Milk Fatty Acid Profile

Table 3 shows that dietary inclusion of increasing levels (10, 20, and 30 g/kg DM) of soybean oil did not affect (*p* > 0.05) the milk concentration of butyric (C4:0), caproic (C6:0), caprylic (C8:0), capric (C10:0), lauric (C12:0), pentadecanoic (C15:0), palmitoleic (C16:1), heptadecanoic (C17:0) and linoleic (C18:2 n-6 cis) fatty acids. However, dietary inclusion of soybean oil decreased (linear effect; *p* ≤ 0.05) the milk concentration of myristic (C14:0) and palmitic (C16:0) fatty acids. In contrast, higher concentrations (linear effect; *p* ≤ 0.05) of stearic and oleic fatty acids were observed in the milk of dairy cows fed soybean oil in their diet.

## 4. Discussion

### 4.1. Milk Yield and Milk Composition

The main results obtained in the current study confirmed that milk production and composition were not affected by the levels of soybean oil tested. However, it was observed that the concentrations of some fatty acids in milk were modified by increasing levels of soybean oil in the diets. Taken together, these findings enabled the study’s objective to be achieved. A recent meta-analysis [14] reported that the inclusion of vegetable oils in dairy cow diets can decrease dry matter intake (DMI) and increase milk yield (MY) in a dose-dependent manner. In the current study, MY and DMI were not affected by the levels of soybean oil (10, 20, and 30 g/kg DM) included in the dairy cow diets. These contradictory effects could be explained by the fact that the doses used in the present study (10–30 g/kg DM) were lower than most of the doses used in the studies in the meta-analysis database by Xin et al. [14]. These results are consistent with those previously reported by other authors [18,31,32,33], who also did not observe significant changes in MY and DMI of dairy ruminants fed increasing levels (20 to 60 g/kg DM) of soybean oil and other vegetable oils in the diet. On the other hand, Castro et al. [34] reported that the inclusion of vegetable oils in ruminant diets can modify the digestibility of dietary mass (DMD), but the effect depends on the amount and fatty acid profile of the vegetable oil used. In the current study, increasing levels of soybean oil in the diet did not affect DMD in dairy cows. Recently, Lock et al. [35] reported that the dietary inclusion of feed ingredients or supplements rich in medium-chain fatty acids (12 and 14 carbons) negatively affects DMD, while products rich in ≥18 carbons have minimal effects on DMD. This would explain the lack of changes observed in DMD in the current study, since most of the fatty acids in soybean oil are long-chain (≥18 carbons) [36].

In the present study, dietary inclusion of increasing levels of soybean oil did not affect the fat, non-fat solids, protein, and lactose content in dairy cows’ milk. Similar to our results, Zheng et al. [18] also did not observe significant changes in the concentration of fat, non-fat solids, protein, and lactose in the milk of dairy cows fed diets containing low levels (21 g/kg DM) of soybean oil. In contrast, other authors [31,32] reported lower milk fat concentrations in dairy goats and cows fed diets containing high concentrations (40–60 g/kg DM) of soybean oil. These results suggest that soybean oil’s effects on milk composition (primarily fat concentration) are dose dependent.

### 4.2. Milk Fatty Acid Profile

Milk composition is influenced not only by rumen metabolism but also by systemic metabolic status and energy balance [37]. In the current study, it was observed that with increasing dose, the response for some fatty acids changed proportionally (linear effect), without any quadratic effects on any response variable. These effects indicate that the linear model captures the trend well, and the quadratic term does not provide additional information for the fatty acid concentrations in the milk evaluated. The safety and origin of feed ingredients are also critical factors influencing animal nutrition and product quality [38]. According to Xin et al. [14], dietary inclusion of vegetable oils can be used as a nutritional strategy to improve the fatty acid profile in ruminant milk. However, in the current study, the milk concentration of C4:0, C6:0, C8:0, C10:0, C12:0, C15:0, C16:1, and C17:0 fatty acids was not affected by increasing levels of soybean oil. Similarly, recent studies [31,39] reported that dietary inclusion of various levels of soybean oil in diets for dairy sheep and goats did not affect the milk concentration of C4:0, C6:0, C8:0, C10:0, C12:0, C15:0, C16:1, and C17:0 fatty acids.

In the present study, lower milk concentrations of C14:0 and C16:0 were observed, while higher milk concentrations of C18:0 and C18:1 n-9 cis were observed in response to the dietary inclusion of high levels (30 g/kg DM) of soybean oil. These effects could have a positive impact on consumer health, since, according to Bello-Pérez and Garnsworthy [6], the intake of milk with a high content of C18:1 n-9 cis and a low content of C14:0 and C16:0 has been associated with a lower incidence of atherosclerosis and cardiovascular disease. Similar to our results, Hamzaoui et al. [31], De Gasperin-López et al. [39], and Dhiman et al. [16] reported lower (−25.9 to −34.2) concentrations of C14:0 and C16:0, as well as higher (+22.9 to +58.7%) concentrations of C18:0 and C18:1 n-9 cis in the milk of dairy ruminants fed increasing levels (20 to 40 g/kg DM) of soybean oil in the diet. Soybean oil has a high concentration of ≥18-carbon-chain fatty acids [38], which decreases the de novo synthesis of C14:0 and C16:0 in the mammary glands of dairy cows [14,35].

On the other hand, Bales and Lock [33] indicate that soybean oil contains a high proportion (around 50%) of C18:2 n-6 cis in its fatty acid profile. A small proportion (between 5 and 40% of the total) of the C18:2 n-6 cis contained in soybean oil can be absorbed and incorporated into ruminant milk [35]. However, most of the C18:2 n-6 cis is used in ruminal biohydrogenation, a process through which C18:2 n-6 cis can form C18:1 n-9 cis as an intermediate and C18:0 as the final product in the rumen [6,15]. A greater formation of C18:1 n-9 cis and C18:0 in the rumen could explain their higher milk concentration observed in the current study, since, according to several authors [1,14], C18:1 n-9 cis and C18:0 are not synthesized in the mammary gland and are only obtained from the ruminal contents. Finally, the current study did not present any limitations in achieving the stated objective. However, emerging precision livestock technologies allow for a more detailed assessment of animal responses to dietary interventions [40].

## 5. Conclusions

In conclusion, dietary inclusion of increasing levels of soybean oil (30 g/kg DM) modifies the fatty acid profile of milk without affecting milk yield or the protein, fat, or lactose content. These findings confirm that soybean oil’s effects on milk fatty acid profile are dose dependent. This is important because it allows dairy cattle nutritionists to use more appropriate ranges of dietary soybean oil. However, it is recommended to evaluate soybean oil doses intermediate to those used in the current study, as well as higher doses, to determine the maximum dose that positively modifies milk fatty acids.

## Figures and Tables

**Table 1:** Composition of dairy cow diets.

	Inclusion of Soybean Oil in the Diet, g/kg DM			
	0	10	20	30
Diets, kg/d				
Oat Straw	4.4	4.4	4.4	4.4
Corn stover	4.4	4.4	4.4	4.4
Commercial concentrate	5.0	5.0	5.0	5.0
Wheat bran	5.0	5.0	5.0	5.0
Soybean oil	0	0.20	0.40	0.60
Chemical composition, % DM				
Dry matter	92.25	92.31	92.41	92.60
Crude protein	12.58	12.84	12.53	12.79
Ashes	5.02	4.93	5.00	4.94
Neutral detergent fiber	49.84	50.02	49.88	49.93
Acid detergent fiber	28.74	28.28	28.22	28.44
Fatty acids provided by soybean oil in diets, g/d				
Palmitoleic (C16:1)	0	10.74	42.97	96.69
Oleic (C18:1 n-9 cis)	0	4.18	16.72	37.63
Linoleic (C18:2 n-6 cis)	0	21.48	85.94	193.37

**Table 2:** Milk yield and composition from dairy cows fed increasing levels of soybean oil in their diet.

	Inclusion of Soybean Oil in the Diet, g/kg DM				SEM	*p*-Value	
	0	10	20	30		Linear	Quadratic
Milk yield (MY), kg/d	14.86	14.61	14.62	14.66	0.17	0.89	0.87
Dry matter intake (DMI), kg/d	18.76	18.74	18.75	18.74	0.008	0.28	0.17
Diet dry matter digestibility (DMD), %	64.01	63.51	63.84	63.00	0.58	0.86	0.29
Chemical composition of milk							
Fat, %	3.02	3.07	3.13	3.21	0.12	0.12	0.52
Nonfat solids, %	8.02	7.73	8.63	8.25	1.66	0.14	0.31
Protein, %	3.27	3.32	3.38	3.39	0.04	0.13	0.37
Lactose, %	4.40	4.36	4.55	4.54	0.06	0.16	0.21

SEM: standard error of treatment means.

**Table 3:** Fatty acid profile in milk from dairy cows fed increasing levels of soybean oil in their diet.

Fatty Acids (FAs), g/100 g of Total FAs	Inclusion of Soybean Oil in the Diet, g/kg DM				SEM	*p*-Value	
	0	10	20	30		Linear	Quadratic
Butyric (C4:0)	4.11	5.22	4.51	5.82	1.17	0.81	0.53
Caproic (C6:0)	0.81	0.84	0.66	0.65	0.08	0.23	0.31
Caprylic (C8:0)	1.27	1.34	1.46	1.55	0.27	0.18	0.32
Capric (C10:0)	2.17	2.28	2.39	2.42	0.32	0.22	0.50
Lauric (C12:0)	3.86	3.53	3.23	3.84	0.67	0.33	0.98
Myristic (C14:0)	11.06 ^a^	10.59 ^a^	8.69 ^b^	7.53 ^b^	0.95	0.04	0.24
Pentadecanoic (C15:0)	1.04	0.91	0.93	0.84	0.07	0.29	0.42
Palmitic (C16:0)	40.86 ^a^	38.68 ^a^	37.39 ^b^	35.68 ^b^	1.22	0.04	0.18
Palmitoleic (C16:1)	1.75	0.95	0.91	1.88	0.35	0.11	0.38
Heptadecanoic (C17:0)	0.51	0.54	0.54	0.58	0.09	0.84	0.87
Stearic (C18:0)	13.17 ^c^	15.04 ^b^	15.68 ^b^	17.14 ^a^	0.69	0.03	0.52
Oleic (C18:1 n-9 cis)	28.43 ^a^	26.6 ^ab^	25.13 ^b^	23.32 ^b^	1.76	0.05	0.68
Linoleic (C18:2 n-6 cis)	1.28	1.43	1.38	1.55	0.34	0.11	0.39

SEM: standard error of treatment means. a, b, c—means within a row with different subscripts differ when *p* ≤ 0.05.

## Data Availability

The datasets used and analyzed during the current study are available from the corresponding author on reasonable request. The data are not publicly available due to restrictions on privacy.

## Abbreviations

The following abbreviations are used in this manuscript:

MY	Milk yield
DMI	Dry matter intake
DMD	Dry matter digestibility
SFA	Saturated fatty acids
PUFA	Polyunsaturated fatty acids
C4:0	Butyric
C6:0	Caproic
C8:0	Caprylic
C10:0	Capric
C12:0	Lauric
C14:0	Myristic
C15:0	Pentadecanoic
C16:0	Palmitic
C16:1	Palmitoleic
C17:0	Heptadecanoic
C18:0	Stearic
C18:1 n-9 cis	Oleic

## References

[1] Godina-Rodríguez J.E., Garay-Martínez J.R., Orzuna-Orzuna J.F., Lara Bueno A., García-García P.A., Vázquez-Silva G. (2025). Milk yield and fatty acid profile in milk of small dairy ruminants supplemented with hemp (*Cannabis sativa* L.) seed products: A meta-analysis. Rev. Chapingo Ser. Zonas Áridas.

[2] Lamanna M., Cavallini D. (2026). Climate narratives and global food security. Vet. Rec..

[3] Graulet B. (2014). Ruminant milk: A source of vitamins in human nutrition. Anim. Front..

[4] Orzuna-Orzuna J.F., Godina-Rodríguez J.E., Garay-Martínez J.R., Reséndiz-González G., Joaquín-Cancino S., Lara-Bueno A. (2024). Milk Yield, Composition, and Fatty Acid Profile in Milk of Dairy Cows Supplemented with Microalgae *Schizochytrium* sp.: A Meta-Analysis. Agriculture.

[5] Tabacco E., Merlino V.M., Coppa M., Massaglia S., Borreani G. (2021). Analyses of Consumers’ Preferences and of the Correspondence between Direct and Indirect Label Claims and the Fatty Acid Profile of Milk in Large Retail Chains in Northern Italy. J. Dairy Sci..

[6] Bello-Pérez E., Garnsworthy P.C. (2013). Trans Fatty Acids and Their Role in the Milk of Dairy Cows. Cienc. Investig. Agrar..

[7] Cavallini D., Lamanna M., Colleluori R., Silvestrelli S., Ghiaccio F., Buonaiuto G., Formigoni A. (2025). The use of rumen-protected amino acids and fibrous by-products can increase the sustainability of milk production. Front. Vet. Sci..

[8] Castillo A.R., Pol M., Gaggiotti M., Di Rienzo J.A., Cavallini D. (2025). Effect of a feed additive based on a mix of condensed and hydrolysable tannins (ByPro^®^) on lactating dairy cows milk production performance. Trop. Anim. Health Prod..

[9] Castillo A.R., Di Rienzo J.A., Cavallini D. (2025). Effect of a mix of condensed and hydrolysable tannins feed additive on lactating dairy cows’ services per conception and days open. Vet. Anim. Sci..

[10] Kokić B., Rakita S., Vujetić J. (2024). Impact of Using Oilseed Industry Byproducts Rich in Linoleic and Alpha-Linolenic Acid in Ruminant Nutrition on Milk Production and Milk Fatty Acid Profile. Animals.

[11] Glasser F., Ferlay A., Chilliard Y. (2008). Oilseed lipid supplements and fatty acid composition of cow milk: A meta-analysis. J. Dairy Sci..

[12] Montazeri E., Riasi A., Ghorbani G.R., Mahyari S.A., Jamali A., Ghaffari M.H. (2024). Effects of pre-and postpartum dietary fat sources (soybean oil versus linseed oil) on lactation performance and blood metabolites in transition dairy cows. Anim. Feed Sci. Technol..

[13] Bayourthe C., Enjalbert F., Moncoulon R. (2000). Effects of Different Forms of Canola Oil Fatty Acids Plus Canola Meal on Milk Composition and Physical Properties of Butter. J. Dairy Sci..

[14] Xin Y., Sun H., Liu S., Lan B., Zhao G., Yin S., Zhao C. (2025). Effects of vegetable oils on feed intake, nutrient digestibility, ruminal parameters, lactation performance, and milk fatty acid composition in cattle: A meta-analysis. Anim. Feed Sci. Technol..

[15] Plata-Pérez G., Angeles-Hernandez J.C., Morales-Almaráz E., Del Razo-Rodríguez O.E., López-González F., Peláez-Acero A., Campos-Montiel R.G., Vargas-Bello-Pérez E., Vieyra-Alberto R. (2022). Oilseed Supplementation Improves Milk Composition and Fatty Acid Profile of Cow Milk: A Meta-Analysis and Meta-Regression. Animals.

[16] Dhiman T.R., Satter L.D., Pariza M.W., Galli M.P., Albright K., Tolosa M.X. (2000). Conjugated linoleic acid (CLA) content of milk from cows offered diets rich in linoleic and linolenic acid. J. Dairy Sci..

[17] Rego O.A., Rosa H.J.D., Portugal P.V., Franco T., Vouzela C.M., Borba A.E.S., Bessa R.J.B. (2005). The effects of supplementation with sunflower and soybean oils on the fatty acid profile of milk fat from grazing dairy cows. Anim. Res..

[18] Zheng H.C., Liu J.X., Yao J.H., Yuan Q., Ye H.W., Ye J.A., Wu Y.M. (2005). Effects of dietary sources of vegetable oils on performance of high-yielding lactating cows and conjugated linoleic acids in milk. J. Dairy Sci..

[19] Gómez-Miranda A., López-González F., Vieyra-Alberto R., Arriaga-Jordán C.M. (2022). Grazed barley for dairy cows in small-scale systems in the highlands of Mexico. Ital. J. Anim. Sci..

[20] NRC (2001). Nutrient Requirements of Dairy Cattle.

[21] AOAC (2019). Official Methods of Analysis.

[22] Van Soest P.J., Robertson J.B., Lewis B.A. (1991). Methods for dietary fiber, neutral detergent fiber and non-starch polysaccharides in relation to animal nutrition. J. Dairy Sci..

[23] Granados-Rivera L.D., Hernández-Mendo O., González-Muñoz S.S., Burgueño-Ferreira J.A., Mendoza-Martínez G.D., Arriaga-Jordán C.M. (2017). Effect of palmitic acid on the mitigation of milk fat depression syndrome caused by trans-10, cis-12-conjugated linoleic acid in grazing dairy cows. Arch. Anim. Nutr..

[24] Candia-López P.A., Hernández-Mendo O., Bárcena-Gama J.R., Améndola-Massiotti R.D. (2025). Protected palmitic acid to mitigate milk fat depression in grazing Holstein cows in the Mexican Highlands. Arch. Anim. Nutr..

[25] Van Keulen J., Young B.A. (1977). Evaluation of acid-insoluble ash as a natural marker in ruminant digestibility studies. J. Anim. Sci..

[26] Domínguez-Peregrino G., González-Garduño R., Ortiz-Pérez D.O. (2024). Physicochemical characterization of milk in 5/8 Holstein x 3/8 Zebu crossbred cows in tropical Mexico. Rev. Colomb. Cienc. Pecu..

[27] SAS (Statistical Analysis System) (2017). SAS/STAT User’s Guide (Release 6.4).

[28] Littell R.C., Henry P.R., Ammerman C.B. (1998). Statistical analysis of repeated measures data using SAS procedures. J. Anim. Sci..

[29] Peretti S., Rosa V.D., Zotti M.L.A.N., Prestes A.M., Ferraz P.F.P., da Silva A.S., Zotti C.A. (2022). Thermoregulation and Performance of Dairy Cows Subjected to Different Evaporative Cooling Regimens, with or without Pepper Extract Supplementation. Animals.

[30] Kaps M., Lamberson W.R. (2004). Biostatistics for Animal Science.

[31] Hamzaoui S., Caja G., Such X., Albanell E., Salama A.A.K. (2021). Effect of Soybean Oil Supplementation on Milk Production, Digestibility, and Metabolism in Dairy Goats under Thermoneutral and Heat Stress Conditions. Animals.

[32] Vieyra-Alberto R., Arriaga-Jordán C.M., Domínguez-Vara I.A., Bórquez-Gastelum J.L., Morales-Almaráz E. (2017). Effect of soybean oil on the concentration of vaccenic and rumenic fatty acids in grazing cow milk. Agrociencia.

[33] Bales A.M., Lock A.L. (2024). Effects of increasing dietary inclusion of high oleic acid soybeans on milk production of high-producing dairy cows. J. Dairy Sci..

[34] Castro T., Manso T., Jimeno V., Del Alamo M., Mantecón A.R. (2009). Effects of dietary sources of vegetable fats on performance of dairy ewes and conjugated linoleic acid (CLA) in milk. Small Rumin. Res..

[35] Lock A.L., dos Santos Neto J.M., de Souza J. (2025). Invited review: Moving from dietary fat to fatty acids—New insights into how fatty acids affect digestibility, metabolism, and performance in dairy cows. J. Dairy Sci..

[36] Kliem K.E., Shingfield K.J. (2016). Manipulation of milk fatty acid composition in lactating cows: Opportunities and challenges. Eur. J. Lipid Sci. Technol..

[37] Magro S., Costa A., Cavallini D., Chiarin E., De Marchi M. (2024). Phenotypic Variation of Dairy Cows’ Hematic Metabolites and Feasibility of Non-Invasive Monitoring of the Metabolic Status in the Transition Period. Front. Vet. Sci..

[38] Gasparini M., Castrezzati G., Cavallini D., Esposito M., Giugliano R., Menotta S., Scortichini G., Ubaldi A., Vitali R., Vivaldi B. (2026). Fipronil presence in feed materials: Hints for a geographical risk analysis to support the transition towards safe and sustainable food systems. Food Addit. Contam. Part A.

[39] De Gasperin-López I., Pinos-Rodríguez J.M., Vicente-Martínez J.G., López-Aguirre S., Estrada-Coates A.T., Contreras-Hernández G. (2025). Effects of CLA, Soybean Oil, and Used Soybean Oil from Fish Friers in Sheep Diets on Milk Lipids and Lamb Tissues. Animals.

[40] Cavallini D., Giammarco M., Buonaiuto G., Vignola G., De Matos Vettori J., Lamanna M., Prasinou P., Colleluori R., Formigoni A., Fusaro I. (2025). Two Years of Precision Livestock Management: Harnessing Ear Tag Device Behavioral Data for Pregnancy Detection in Free-Range Dairy Cattle on Silage/Hay-Mix Ration. Front. Anim. Sci..

